# A Comparative Study of Knee Joint Proprioception Assessment in 12-Week Postpartum Women and Nulliparous Women

**DOI:** 10.7759/cureus.48101

**Published:** 2023-11-01

**Authors:** Grisha R Ratnani, Shubhangi Patil, Pratik Phansopkar, Nikita S Deshmukh

**Affiliations:** 1 Department of Community Health Physiotherapy, Ravi Nair Physiotherapy College, Datta Meghe Institute of Higher Education and Research (DU), Wardha, IND; 2 Department of Musculoskeletal Physiotherapy, Ravi Nair Physiotherapy College, Datta Meghe Institute of Higher Education and Research (DU), Wardha, IND

**Keywords:** fall, nulliparous, postpartum, proprioception, the knee joint

## Abstract

Introduction

Proprioception is one's capacity to perceive bodily position, alignment, and movement. Several connective tissues, such as skin, ligaments, joint capsules, and muscles in the body, contain proprioceptive sensory receptors. Joint elasticity results from hormonal variations, notably the peak relaxin hormone during pregnancy, which also affects proprioceptive receptors. The musculoskeletal system may be affected by hormones and anatomical changes brought on by pregnancy, including joint laxity and modifications to posture and gait. The capacity to perceive the joint position and movement, or proprioception, may be impacted. To comprehend the impacts of pregnancy on joint function and postpartum women's rehabilitation options, this study compares knee joint proprioception in women who gave birth 12 weeks ago to nulliparous women. The study aims to assess and compare the degree of alteration in knee joint proprioception in 12-week postpartum females.

Methodology

A total of 160 participants were assessed during the entire study. Women from 18 to 35 years of age were included in the study. Women with any present knee joint injury, multiparty, or relevant surgical history were excluded. The procedure was performed under the author's surveillance at the Department of Community Health Physiotherapy. The knee joint reposition test was used to assess the knee joint proprioceptive error among two groups (80 each), including nulliparous women and the other 12-week postpartum women. An image tool provided by the University of Texas Health Science Centre at San Antonio (UTHSCSA) was created and offers the tool as computer software or a digital application for handling medical pictures and associated data, software 3.0 was used to determine the angular variation between angles in the targeted and achieved positions during the test.

Result

A significant proprioceptive error was observed among 12-week postpartum women compared to the nulliparous group of women. The mean error of knee joint repositions among 12-week postpartum women was 0.80±6.08 (P=0.0001), and among nulliparous women was 0.09±0.72 (P=0.0001).

Conclusion

Concluding insight that pregnancy affects postpartum women's risk of fall injuries and joint function due to altered proprioception. Compared to nulliparous women, proprioceptive error for the dominant knee joint was significant among 12-week postpartum females. The hormonal changes during pregnancy affect the proprioceptive receptors, especially the relaxin hormone surge, which results in joint laxity and may impair joint position sensing, increasing the risk of falls. To better acknowledge the effects of pregnancy on joint function and postpartum women's rehabilitation options, this study compares knee joint proprioception in postpartum and nulliparous women. It proves right about altered proprioception post-childbirth. The results of this study might aid medical practitioners in creating successful rehabilitation plans and treatments to stop postpartum women from falling.

## Introduction

One's capacity to perceive bodily position, alignment, and movement is known as proprioception. In response, proprioception is commonly referred to as the sense of joint position, kinesthesia, movement sensation, sense of effort, and sense of force. Along with pain, tactile, and thermal sensation, kinesthesia is a part of the bodily sensory system. It is known as an interceptive system because its sensory input originates from changes to internal structures [1]. Several connective tissues, such as skin, ligaments, joint capsules, and muscle tissue throughout the limbs, trunk, and neck, contain proprioceptive sensory receptors. Capsular, ligamentous, and cutaneous mechanoreceptors are thought to complement spindle input for position and movement awareness throughout most motions, with the muscle spindles being the primary source of proprioceptive information [2].

During the period of pregnancy, the female body goes through numerous hormonal and anatomic modifications that have an impact on the musculoskeletal system. These changes could cause musculoskeletal disorders, raise the risk of injury, or change the course of existing conditions [3]. Pregnancy affects the soft tissues, joints, posture, and gait. Joint laxity develops during pregnancy due to hormonal fluctuations, which lasts even beyond six weeks postpartum [4]. Hormonal alterations throughout the pregnancy have been shown to affect the equilibrium of labyrinthine fluids, which has a straightforward impact on the enzyme process and neurotransmitter activity. Relaxin hormone is increased to its peak during pregnancy, resulting in increased laxity. Raised levels of estrogen and relaxin hormones affect the neuromuscular system and alter the mechanics of tendons and ligaments where proprioceptive receptors lie [5]. Variations in a childbearing woman's static stability may be caused by weight gain during pregnancy and its uneven distribution, especially in the anterior belly area, reconciling posture modifications essential for the anteroposterior center of gravity position readjustment and increased joint laxity [6]. Prevalent alterations in the body during pregnancy involve knee hyperextension, collapsing of the medial longitudinal foot arch, anteriorly tilting the pelvis, lordosis of lumbar curvature, and gaining volume, length, and breadth of the foot [7].

A 27% fall rate has been observed throughout pregnancy, particularly around the third trimester, due to a decrease in maintaining the balance that lasts six to eight weeks after childbirth. The loss of proprioception in pregnant women has not been examined, even though balance difficulties and visual dependency have been reported. Proprioception is considered the sixth sense of the human body. During pregnancy, fluctuation in hormonal levels reaches its peak, and relaxin hormone is one of them, which causes laxity of ligaments joints, resulting in a lack of proprioception. Proprioception dysfunction in the lower extremities, significantly in the knee and mortise joints, is one of the major causes of a higher risk of fall injuries. A previous investigation on knee proprioception revealed literature indicating that knee proprioception changes during pregnancy [8]. It is unclear whether these alterations revert to the baseline during the postpartum period. As a result, we wanted to investigate knee joint proprioception in women 12 weeks after the delivery and compare it with the knee joint proprioception of non-pregnant women. As a result, this research is being done to compare the lack of proprioception in women after 12 weeks of delivery and women who are not pregnant.

## Materials and methods

After receiving ethical authorization and approval from the Institutional Ethical Committee of Datta Meghe Institute of Higher Education and Research(DU) with Ref. No. (DMIMS(DU)/IEC/2022/919). This research was conducted in local communities and Acharya Vinobha Bhave Rural Hospital, Sawangi, Wardha, Maharashtra. The study randomly chose 80 women in each group aged between 18 and 35. One group belongs to women in the 12th week of postpartum, and the other includes nulliparous females. Women with any knee contracture or deformity, a history of arthritis, instability in the knee joint, a history of knee surgery, or any neurological disorders were excluded from participating in the research. The following process was conducted on 80 age-matched nulliparous women with regular menstrual cycles.

Procedure

**Figure 1:**
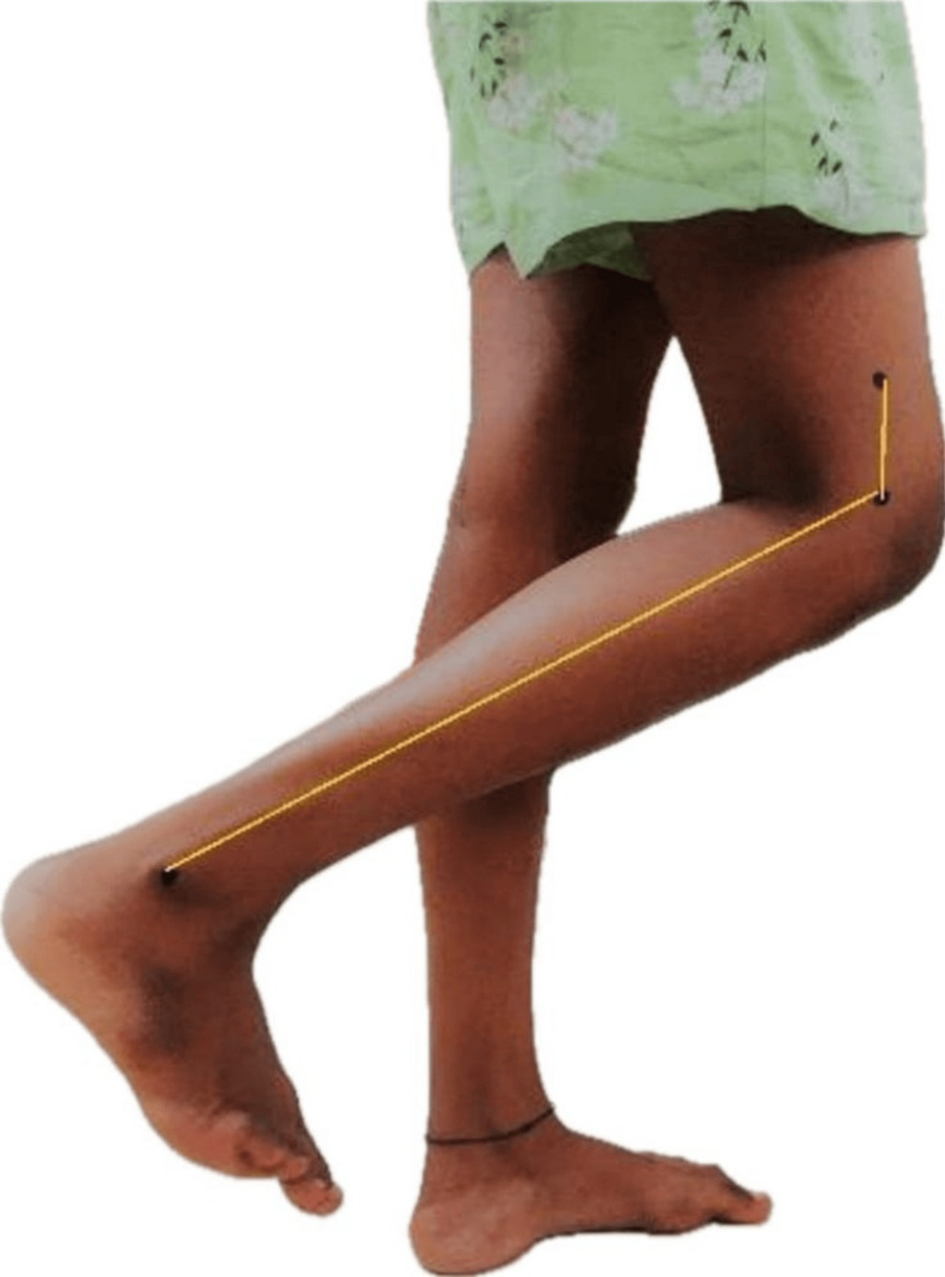
Knee joint reposition test in a nulliparous subject

**Figure 2:**
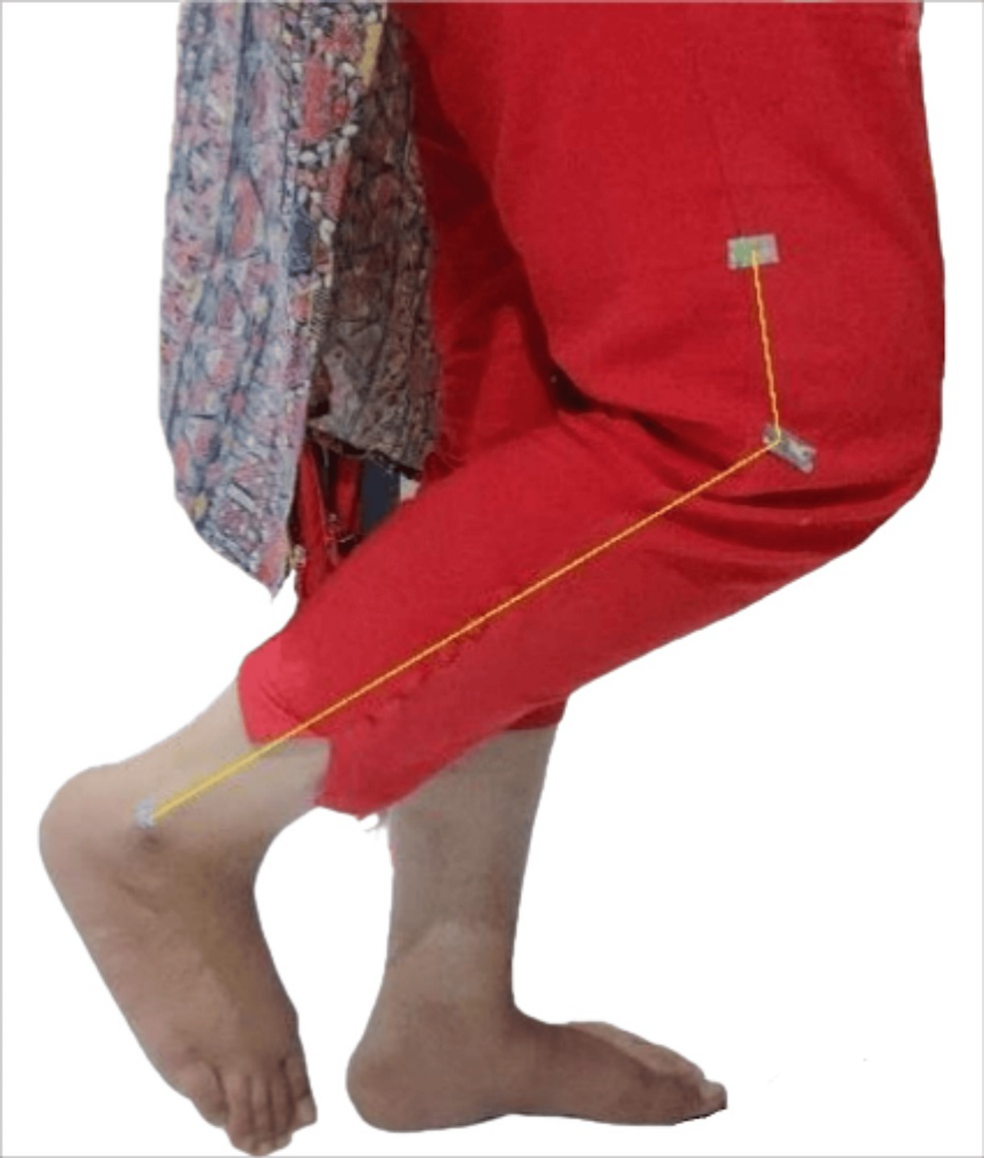
Knee joint reposition test in 12-week postpartum subject

All of the individuals provided their informed consent. The subjects were instructed to sustain their weight by standing against a wall with no more than two fingers of support. The markers were positioned 5 cm above the lateral femoral condyle, at the lateral malleolus, and at the lateral femoral condyle of the dominant lower extremity. The individuals were asked to keep their eyes closed. The knee was tested by flexing, extending ten times, and attaining a specific position. This position was regarded as the ideal angle. The subjects were asked to stay in the same position for 15 seconds and remember it. A footstool with an advanced high-pixel camera perpendicular to the knee was placed 60 cm from the subject's feet. The desired position was captured on camera. Afterward, the patients were instructed to move their knee 10 times in full flexion and extension before positioning it at the desired angle. Once more, this position was captured, and the photos were uploaded to a computer. The University of Texas Health Science Centre at San Antonio (UTHSCSA) image tool 3.0 was used to assess the images and determine the difference between the original and final angles, as seen in Figures 1, 2. The UTHSCSA developed and rendered available software or a digital application for collecting, processing and maintaining medical pictures and related data. It was recorded and taken to examine the reposition error-the difference between the initial and final angles. According to the researchers, the knee joint reposition test's reliability is 0.81 [9]. The knee joint reposition test post-evaluation in a nulliparous subject is shown in Figure 1, and 12-week post-partum women in Figure 2.

Data analysis

Students' paired and unpaired t-tests were employed in the statistical analysis using descriptive statistics to summarize and describe the obtained data and inferential statistics to draw the conclusion. The analysis was conducted using the SPSS 27.0 version (IBM Corp., Armonk, NY) of the software, and a significance threshold of p<0.05 was utilized.

## Results

**Figure 3:**
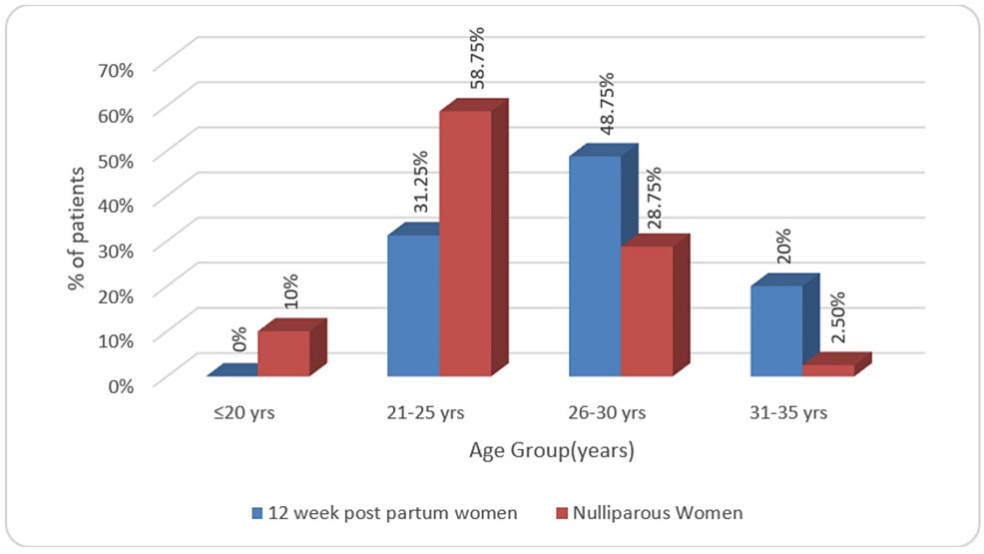
Graphical presentation of distribution of volunteers in two groups according to their age in years

The subjects were distributed among two groups according to respective age groups as shown in Figure 3.

**Figure 4:**
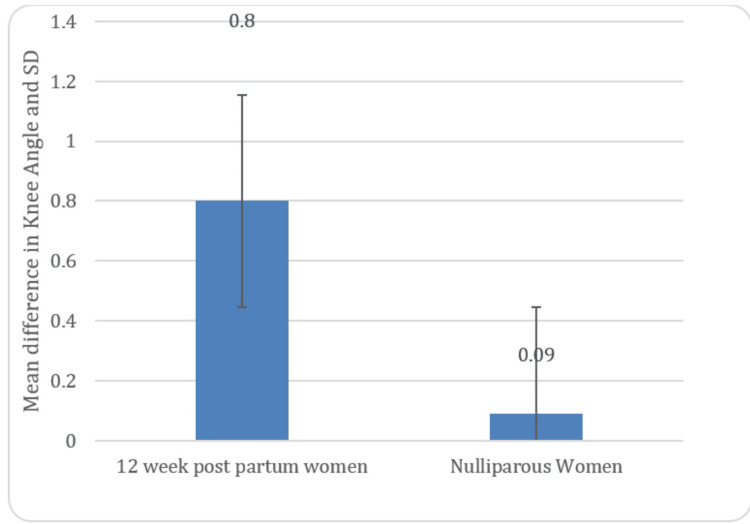
Graphical presentation of Comparison of knee angle in two groups throughout the repositioning test

In Table 1, the mean error of knee joint repositions among 12-week postpartum women noted was 0.80±6.08 and among nulliparous women noted was 0.09±0.72 present in Figure 4.

**Table 1:** Comparison of knee angle in two groups at target angle and achieved angle

Group	Targeted angle	Achieved angle	Mean Difference in position error	Student’s paired t-test
12-week postpartum women	66.57±22.82	67.37±23.44	0.80±6.08	1.17 P=0.24,NS
Nulliparous Women	96.49±22.07	96.58±22.09	0.09±0.72	116 P=0.24,NS
Comparison between two groups(Student’s unpaired t-test)				
t-value	8.42 P=0.0001,S	8.11 P=0.0001,S		

All the measures post the study stated that there is marked proprioceptive error among women in their 12th week of postpartum, i.e., 0.80±6.08 (P=0.24) whereas in the nulliparous group of women, a minimal proprioceptive error was seen 0.09±0.72 (P=0.24).

## Discussion

In this study, two groups, including 80 participants each, one involving post-partum women in their 12th week and other nulliparous women all belonging to the age group 18-35 years, were assessed and reported a significant proprioceptive error even after 12 weeks of delivery when compared to nulliparous women of the same age group. Increased 20% of one's body weight throughout pregnancy elevates the stress on the primary joints of lower extremities, which may persist even after delivery, which can affect a woman's quality of life. In a study, Ritchie et al. claimed that soft tissue swelling is among the major physiological variations during pregnancy. Eighty percent of childbearing women experience oedema at some point throughout their pregnancy, leading to musculoskeletal disorders that stay even after the delivery. The musculoskeletal system is considerably affected during pregnancy, which might result in novel injuries or reduce the threshold for many prevalent disorders [10]. Blecher et al. concluded that the hormonal changes during pregnancy are responsible for a transitory increase in laxity of the ACL of the knee joint, which is typically observed in patients after one year following ACL reconstruction [11]. Gupta et al. in a study assessed 36 pregnant ladies throughout all trimesters of the antenatal period and recorded proprioception of the knee joint. She used a digital inclinometer for assessment. It concluded that lots of changes take place during pregnancy, causing curtailing of knee joint proprioception and increasing the risk of falls [4]. Li et al. worked on a project in which they assessed proprioception of the knee joint in 30 young men using three different methods: joint angle reset, motion minimum threshold measurement, and force sense reproduction [12]. Ramachandra et al. stated that ankle proprioception was considerably impacted during pregnancy in the last trimester and did not revert to baseline even six weeks post-childbirth. It briefed that proprioceptive input from the ankle joint is important for maintaining postural stability. It is well established that the ankle plantar flexors are crucial in maintaining postural stability in pregnant women. The study involved 70 pregnant women assessed during their third trimester and six weeks postpartum for ankle joint repositioning and kinesthetic sense. It concluded that ankle joint proprioception was altered and did not recover after six weeks postpartum. This study successfully assessed the proprioceptive error of knee joints among postpartum women even after 12 weeks of delivery compared to nulliparous women of similar age groups [13].

The study's findings indicate that women who are 12 weeks postpartum have much higher proprioceptive inaccuracy than women who are not pregnant or lactating. This might be owing to the altered proprioceptive input gained from the lax ligaments around the knee joint. It has been shown that relaxin hormone levels rise 10 times faster during pregnancy, predisposing to ligament and joint laxity, which may compromise the receptors' capacity to detect movement [14].

Physical therapy rehabilitation must be begun in the second and third trimesters and continued even after delivery for early recovery of all the physiological disturbances that occurred throughout the period. These interventions may include proprioceptive training, balance training, tai chi exercises, etc., which benefit childbearing women the most and are safer for them [15]. To lower the rate of falls caused by postural instability, this study supports the need for proprioceptive training programs in pregnant women, especially immediately following delivery.

The results only broadly apply to primiparous women in the research area who are 12 weeks postpartum. Furthermore, future research is required for multiparous women who needed a different study design and were not included in the current investigation.

## Conclusions

The study has illuminated the mysterious connection between pregnancy and postpartum alterations in knee joint proprioception. According to the results, proprioceptive alterations in postpartum women are retained even after 12 weeks of delivery. This research proves that the rising incidence of falls among postpartum women can be caused due to alterations in knee joint proprioception. The effects of hormonal and musculoskeletal modifications go beyond the knee joint, possibly affecting the functioning of the entire musculoskeletal system. This study highlights how crucial it is to do more research to fully comprehend these alterations and their long-term consequences, with possible implications for treatment and prevention strategies. To promote the best possible musculoskeletal health and general well-being, healthcare practitioners must be aware of these changes and adapt treatment and exercise regimens accordingly, especially for postpartum women. The study also emphasizes the need for proactive healthcare approaches that target the special requirements of postpartum women in order to improve their physical recovery and quality of life.
